# Transactions of the American Gynecological Society

**Published:** 1891-09

**Authors:** 


Transactions of the American Gynecological Society. Vol. XV. For the year 1890. Small octavo, pp. xxxix.—411. Philadelphia : William J. Dornan. 1890.

This volume of the Transactions of this distinguished Society is a record of the work it performed in Buffalo last year. The papers are for the most part of a high order of merit, and the discussions are interesting and pointed. Perhaps one of the strongest papers in the book is that of Dr. J. M. Baldy, on the Diagnosis and Treatment of Early Ectopic Gestation, and another by Dr. Ed. W. Jenks, on the Resemblance of Some Forms of Benign Disease to Malignant, deserves special mention; but it is a volume that must be seen to be appreciated, and read to be understood. We may affirm, without fear of exaggeration, that it is a book that necessarily must be found on the shelves of every progressive man who teaches or practises gynecology at the present day.

